# Genome communication in plants mediated by organelle–n­ucleus-located proteins

**DOI:** 10.1098/rstb.2019.0397

**Published:** 2020-05-04

**Authors:** Karin Krupinska, Nicolás E. Blanco, Svenja Oetke, Michela Zottini

**Affiliations:** 1 Institute of Botany, Christian-Albrechts-University of Kiel, Olshausenstraße 40, 24098 Kiel, Germany; 2 Centre of Photosynthetic and Biochemical Studies, Faculty of Biochemical Science and Pharmacy, National University of Rosario (CEFOBI/UNR-CONICET), Rosario, Argentina; 3 Department of Biology, University of Padova, Via U. Bassi 58B, 35131 Padova, Italy

**Keywords:** dual localization, genome communication, mitochondria, nucleus, organelles, plastids

## Abstract

An increasing number of eukaryotic proteins have been shown to have a dual localization in the DNA-containing organelles, mitochondria and plastids, and/or the nucleus. Regulation of dual targeting and relocation of proteins from organelles to the nucleus offer the most direct means for communication between organelles as well as organelles and nucleus. Most of the mitochondrial proteins of animals have functions in DNA repair and gene expression by modelling of nucleoid architecture and/or chromatin. In plants, such proteins can affect replication and early development. Most plastid proteins with a confirmed or predicted second location in the nucleus are associated with the prokaryotic core RNA polymerase and are required for chloroplast development and light responses. Few plastid–nucleus-located proteins are involved in pathogen defence and cell cycle control. For three proteins, it has been clearly shown that they are first targeted to the organelle and then relocated to the nucleus, i.e. the nucleoid-associated proteins HEMERA and Whirly1 and the stroma-located defence protein NRIP1. Relocation to the nucleus can be experimentally demonstrated by plastid transformation leading to the synthesis of proteins with a tag that enables their detection in the nucleus or by fusions with fluoroproteins in different experimental set-ups.

This article is part of the theme issue ‘Retrograde signalling from endosymbiotic organelles’.

## 1. Introduction

All eukaryotic cells have in addition to the nuclear genome a small genome in mitochondria encoding only a minute part of the organelle's proteome*.* The plant cell has a third genome in plastids which in higher plants encodes about 85 proteins. Mitochondria and plastids are endosymbiotic organelles with prokaryotic ancestors. The majority of the prokaryotic genes were either lost or transferred to the nucleus during evolution [1], and most of the organelle proteome are nuclear-encoded. Organelles have multiple copies of their small genomes; the number per cell varies with respect to development and environmental cues [2]. Organelle genomes are organized in compact nucleoprotein structures, called nucleoids, which contain proteins involved in gene expression such as RNA polymerases, transcription factors, and DNA architectural binding proteins [3–7] as well as some unexpected proteins with roles in the metabolism of the organelles [4]. In humans, the mitochondrial DNA (mtDNA) copy number varies between 100 and 1000 per cell depending on the type of tissue. Compared with the small compact mtDNA of animals, mtDNA in plants is larger and more variable, coinciding with a higher recombination frequency [2,8]. Plant mitochondria contain fewer copies of mtDNA than animal mitochondria, indicating that some mitochondria have no DNA [2]. The plastid DNA (ptDNA) copy numbers are much higher than the mtDNA copy numbers and vary in chloroplasts from a few up to thousands per cell [9].

During the evolution of eukaryotes, the major DNA architectural proteins typical for bacteria such as the HU protein have been replaced with eukaryotic proteins for packaging of DNA [10]. In contrast to animals, where the major DNA-binding protein in mitochondria (TFMA) binds to double-stranded DNA, plant mitochondria possess a variety of single-stranded DNA-binding proteins which have been proposed to function as transcriptional modulators [5]. It is likely that the eukaryotization of nucleoids is linked to a tighter coordination of gene expression in the different compartments.

A coordinate expression of the different genomes is essential for development and adaptation to the environment. Organelles are the powerhouses of the cell, producing ATP in conjunction with electron transfer reactions which are extremely sensitive to environmental change. Efficient operation of energy-producing reactions in both organelles is of fundamental importance for the energy supply of the organism and for stress avoidance. The organelles are sensors of environmental change, while the nucleus responds to functional disturbances in the organelles by changes in gene expression. The essential communication between the nucleus and the two DNA-containing organelles is mediated by anterograde and retrograde signalling [11–15].

Conceptually, the translocation of proteins from organelles to the nucleus is the simplest and most direct way of retrograde communication [16]. Indeed, several DNA-binding proteins in plastids have a second localization in the nucleus [4]. The balance between organelle and nuclear pools of these organelles can be altered by two principal mechanisms, i.e. dual targeting to either organelles or nucleus from the cytoplasm or import into organelles and subsequent relocation to the nucleus [17].

In this review, all proteins with dual localization in either one of the organelles or the nucleus will be designated as organelle–nucleus (ON) proteins or more specifically as either mitochondria–nucleus (MN) or plastid–nucleus (PN) proteins. Identical ON proteins relocated from organelles to the nucleus will be called echoproteins [18,19]. This term does not apply to those ON proteins dually targeted to organelles and nucleus, because their nuclear forms are usually larger owing to the presence of an N-terminal organelle target peptide (OTP, either MTP (mitochondrial target peptide) or PTP (plastid target peptide)). Often, organelle-located proteins have similar or context-related functions in the two compartments where they are present. To avoid misinterpretations, the term ‘moonlighting’, characterizing a protein with different unrelated functions [20], will not be applied to this category of proteins.

In the focus of this review are plant proteins with functions linked to coordination of the different genomes and their activities. The different mechanisms of their subcellular distribution will be discussed in comparison with the knowledge obtained with non-photosynthetic organisms. In addition, methodological approaches for analyses of protein movements from organelles to the nucleus will be presented.

## 2. The significance of dual-localized proteins in non-plant eukaryotic cells

Mitochondria are the major powerhouses of non-photosynthetic organisms. The energy, in the form of ATP, is generated by oxidative phosphorylation (OXPHOS) and provided for numerous cellular activities. In many common human diseases, disturbances in mitochondrial metabolism linked with oxidative stress affect mtDNA that encodes central proteins required for OXPHOS. Thereby energy production is reduced. Owing to its implications for human health, research on the regulation of mitochondrial activities involving MN proteins is of increasing importance. Many of the MN proteins were originally overlooked in their second compartment, because they localize predominantly to one compartment and have a rather small pool in the second one [21]. Progress in the methodology for detection of low-abundance proteins during the past 20 years has enabled the identification of numerous proteins in cellular compartments other than those originally attributed to them [21]. The regulated balance between the two protein pools could be relevant for the biological function of MN proteins [22].

### (a) Nuclear transcription factors with a secondary activity in mitochondria

**Figure 1.**
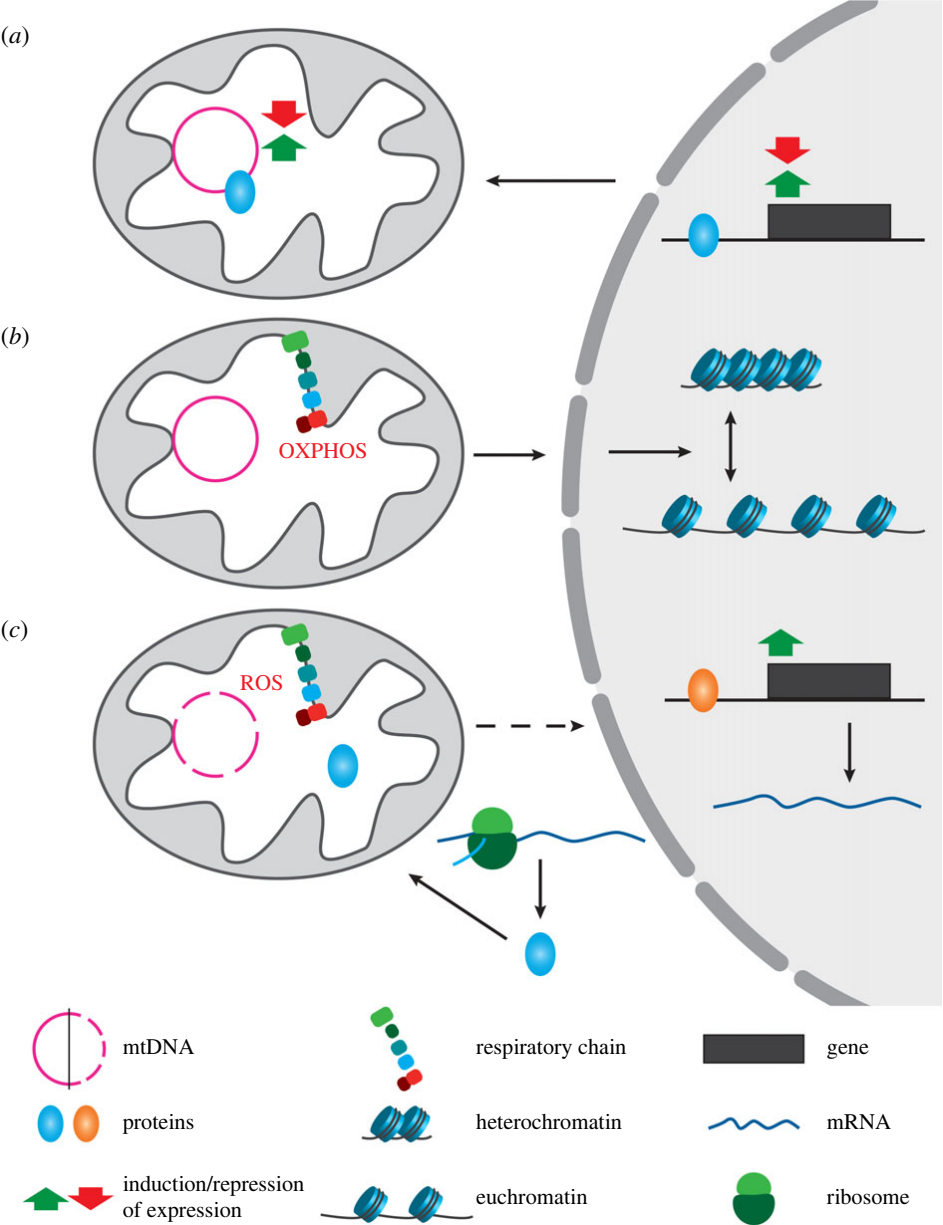
Schematic of three different categories of MN proteins in eukaryotic cells: nuclear transcription factors with a role in mitochondrial gene expression such as ATFS-1 (*a*), mitochondrial enzymes such as fumarase with a second role in the nucleus that is associated with chromatin remodelling (*b*), and proteins involved in MN crosstalk related to DNA damage and repair, e.g. PARP1 (*c*).

Several mammalian nuclear transcription factors locate in addition to mitochondria where they act directly as regulators of mitochondrial gene expression (figure 1*a*), e.g. the tumour suppressor p53 and the thyroid hormone receptor T_3_ (p43) [23], the activating transcription factor associated with stress-1 (ATFS-1) [24,25] and the MOF transcription factor, which belongs to the MYST family of acetyl transferases. MOF resides in mitochondria, and nucleus together with some of its interacting proteins, and regulates OXPHOS by controlling the expression of respiratory genes from both nuclear and mtDNA [26]. By *in organello* analyses and mitochondria-specific overexpression, the mitochondrial role independent of the nuclear role of several transcription factors has been investigated [23]. Often, the same signals that regulate the activity of the nuclear pool also regulate the activity of the mitochondrial pool [23], allowing coordinate changes in gene expression.

### (b) Mitochondrial proteins with a secondary localization in the nucleus

By contrast, an increasing number of mitochondrial proteins have been reported to have a second localization in the nucleus [22]. Among these proteins are several enzymes of the tricarboxylic acid (TCA) cycle involved in the metabolism of mitochondria, which in the nucleus adjust gene expression in response to metabolic flux (figure 1*b*). A typical example is yeast fumarase which is the first metabolic enzyme described to have a second localization in the nucleus. In the mitochondria, it converts fumarate to malate and in the nucleus it participates in DNA repair [27]. Upon ionizing radiation provoking DNA double-strand breaks in the nucleus, a part of the pool is rerouted to the nucleus, where it displays a DNA-damage response by inhibiting histone demethylation at double-strand breaks [28].

Another well-studied example is the pyruvate dehydrogenase complex (PDC), which oxidatively decarboxylates pyruvate to form NADH and acetyl-CoA in both compartments. While in mitochondria acetyl-CoA is required for the TCA cycle, in the nucleus it is used for histone acetylation [29,30]. The subunits of PDC lack MTPs and were detected in the nucleus even in the presence of translational inhibitors [29] (§5), indicating that PDC is an echoprotein complex which despite its large size (70–100 nm) translocates to the nucleus as an intact particle.

It is intriguing that, besides these metabolic enzymes, several other proteins with predominant localization in mitochondria are involved in the maintenance of nuclear and mitochondrial genome integrity [22]. Among these is the major transcription factor A of mitochondria (TFAM), which is responsible for transcription initiation linked with a bending of mtDNA [31]. In the nucleus, it was shown to change gene expression in response to mitochondrial metabolism [32]. Other proteins with similar distribution are components of telomerase (TERT, telomerase reverse transcriptase), the TERT1-interacting protein 2 (TIN2) and the DNA helicase RECQ helicase-like 4 protein (RECQL4). TERT is predominantly imported into mitochondria, where it protects the genome [33]. It contains a bipartite nuclear targeting signal that regulates its shuttling in and out of the nucleus, and an MTP that guides a fraction of TERT to the mitochondrial matrix [34,35].

Two MN proteins, recently identified and important for human health, are CHCHD2 (coiled coil–helix–coiled coil–helix2) and CHCHD10 [36,37]. They belong to the family of twin CX_9_C motif proteins which are targeted to the mitochondria intermembrane space, where they affect biogenesis of cytochrome *c* oxidase and protein import [38]. The proteins were shown to regulate OXPHOS directly in mitochondria by binding to cytochrome *c* oxidase, and indirectly in the nucleus, where they act as regulators of genes encoding proteins of the mitochondrial metabolism, including their own genes [39–41]. Owing to its dual role in the regulation of mitochondrial function, CHCHD2 has been also dubbed mitochondrial nuclear retrograde regulator 1 (MNRR1) [39].

### (c) Proteins involved in MN crosstalk related to DNA damage

Reactive oxygen species (ROS) produced as by-products of OXPHOS can cause damage to mtDNA. Owing to its proximity to the site of OXPHOS, mtDNA is likely more susceptible to oxidized DNA damage than nuclear DNA. If mtDNA is not repaired immediately, heteroplasmy (co-existence of undamaged and damaged mtDNA) might occur, and OXPHOS decreased in consequence [42]. Prioritized protection of the mitochondrial genome might prevent a further increase in the level of ROS and indirectly safeguard the nuclear genome, which eventually would get damaged if the mutated mtDNA stayed unrepaired. Mitochondria can combat DNA damage by repair mechanisms that are analogous to those found in the nucleus [42,43]. Among the proteins involved in the repair of mtDNA in mammals are two proteins with a dual localization in mitochondria and nucleus, i.e. POLY (ADP ribose) polymerase 1 (PARP1) and p53. In mitochondria, PARP1 is involved in the detection of single-strand breaks and in the nucleus it regulates expression of genes encoding other proteins involved in DNA repair [44] (figure 1*c*).

## 3. Organelle–nucleus-located proteins in the plant cell

As early as 1998, Small and co-workers postulated that, among the enzymes involved in DNA maintenance and gene expression, several might be targeted to all DNA-containing compartments in order to guarantee the integrity of the genomes [45]. This is particularly important for organelle genomes, which are constantly subjected to changing environmental conditions [2]. Among the proteins predicted to be dual-located in organelles and nucleus [46] are several transcription factors of eukaryotic origin [47]. These contain motifs typical for nuclear transcription factors, such as zinc finger motifs in, e.g., the CND41 protease, leucine zipper motifs such as in, e.g., the PEND protein and basic helix–loop–helix motifs as in, e.g., NtWIN4 [48]. It has been suggested that clustered basic residues at the N-terminus might constitute a key structure for the conversion of these proteins from nuclear transcription factors to organelle-resident proteins [48].

### (a) MN proteins

The MN proteins identified in non-photosynthetic organisms are not necessarily dual-located in plants. For example, while fumarase is located in nuclei of yeast, there is no indication for nuclear localization in plants. By contrast, dihydrofolate reductase-thymidylate synthase (DHFR-TS) has been demonstrated in nuclei of animal cells as well as in those of plants [49].

Ligase1. In *Arabidopsis*, DNA ligase 1 (AtLIG1) provides the major DNA ligase activity in cells and plays a key role in both DNA replication and excision repair pathways. The AtLIG1 protein contains an MTP and a nuclear localization sequence (NLS). The translation of different isoforms and their targeting is regulated during plant development, when the relative importance of DNA ligase activities in the nucleus and cellular organelles can change depending upon cell type and the cell's metabolic state [50].

PPR protein localized to the nucleus and mitochondria 1 (PMN1)*.* *PNM1* encodes a novel pentatricopeptide repeat protein. In mitochondria, PNM1 is associated with polysomes and may play a role in translation. In the nucleus, PNM1 interacts with the transcription factor TCP8, which can bind to the promoter of *PNM1*. This suggests that PNM1 is involved in the autoregulation of its own gene [51].

Prohibitin 3 (PHB3) belongs to the highly conserved family of prohibitins forming ring-like complexes in mitochondria that were proposed to lead to a functional compartmentalization in the inner membrane [52]. PHB3 controls ROS homeostasis in mitochondria and thereby regulates cell division and root development [53]. Furthermore, root development depends on the impact of nuclear PHB3 on genome stability, DNA repair and replication [54]. PHB3 translocation to the nucleus links operational information about mitochondria with mechanisms that control DNA-damage response and cell cycle [55]. The role of the nuclear fraction of PHB3 in DNA-damage response was demonstrated successfully by hemicomplementation of *phb3* mutants with a version of PHB3 lacking an N-terminal nuclear export sequence (NES) [54].

Opener (OPNR) is expressed in rapidly dividing cells and might function in cell cycle progression. Loss of function results in embryo lethality. Intriguingly, OPNR localizes to the nuclear envelope and mitochondria [56]. In the inner nuclear envelope it interacts with SUN1, a transmembrane protein that is involved in nuclear DNA-damage responses [57,58]. In mitochondria, it co-localizes with PHB3/4, suggesting a functional relationship between these proteins.

Sirtuin1 and 2 (SRT1, 2) play a relevant role in fine tuning of mitochondrial energy metabolism [59,60]. Genetic and transcriptome analyses revealed that SRT1 and SRT2 are furthermore required for negative regulation of certain ethylene-responsive genes. They have a histone deacetylase activity and interact in the nucleus with positive regulators of ethylene signalling, thereby maintaining a low level of acetylated histones at genes repressed by ethylene [61].

Thioredoxin (TRXO). So far, the presence of a dual-located TRX isoform (PsTRXO1) has been reported only in pea (*Pisum sativum*). In mitochondria, PsTRXO1 in involved in the regulation of the activity of ALTERNATIVE OXIDASE. In nuclei, it has been suggested to protect the genome against oxidation and to control the transcription of non-coding DNA [62].

Dihydrofolate reductase-thymidylate synthase (DHFR-TS) catalyses the penultimate step in folate biosynthesis. The three isoforms in *Arabidopsis* are localized in mitochondria, cytosol and nucleus, and expression and localization depend on the tissue. The third isoform, which inhibits the activity of the two others, has in addition to its mitochondrial localization a nuclear localization, but not in the same cell [49]. It has been proposed that nucleus-located DHFR-TS could take part in the formation of the replicase complex as its animal counterpart [49].

DHFR-TS, SIR and PHB3 are also, in animals, located in mitochondria and the nucleus. DHFR-TS localization varies depending on the type of tissue. Owing to their conservation in all eukaryotic organisms [55], PHB proteins provide an excellent model to study the evolution of interorganellar translocation [63].

### (b) PN proteins

Several proteins with a dual localization in plastids and nucleus have previously been described in a review [64]. In the electronic supplementary material, table S1, these and more recently discovered examples are listed. Usually, the functionalities of these proteins have been associated with DNA. Most of the PN proteins play roles in either chloroplast development, stress management or the coordination of organelle division and cell cycle. Several proteins can, however, not be assigned to any of these categories, so far. For some of them only the function in one of the two compartments is known, e.g. ANN5 (annexin5), ADT5 (arogenate dehydratase 5), PEND (plastid envelope DNA-binding protein) and MFP1 (MAR attachment region-binding filament-like protein 1).

#### (i) Proteins required for chloroplast development

Plastids have a complex transcriptional apparatus consisting of plastid- and nucleus-encoded proteins. A phage-type RNA polymerase related to the mitochondrial RNAP is nucleus-encoded (NEP, nucleus-encoded RNA polymerase), while the core subunits of a prokaryotic RNAP are plastid-encoded (PEP, plastid-encoded RNA polymerase). Chloroplast development is associated with a shift from NEP-based transcription to PEP-dominated transcription in plastids (for a review, see [65]). The activity of PEP requires nuclear-encoded sigma factors and a couple of eukaryotic proteins associated with it (PAPs, PEP-associated proteins), indicating an evolutionary re-shaping of the prokaryotic core machinery [66].

HEMERA (pTAC12, PAP5). Originally, HEMERA was identified as a component of the transcriptionally active chromosome and hence dubbed pTAC12 [67]. It was found to bind to RNA and ssDNA [68], and was also named PAP5 as a component of the PEP complex [69]. In the nucleus, the protein was found to be important for phytochrome B localization to photobodies and for degradation of phytochrome-interacting factors (PIFs), which leads to photomorphogenesis [70,71]. Moreover, it is required for PIF4-dependent induction of temperature responses [72]. By immunoblot analyses, the forms of HEMERA in *Arabidopsis* and maize were shown to have the same molecular weights in plastids and in the nucleus [73,74]. Full complementation of the *Arabidopsis* *hmr* mutant and nuclear localization required the presence of a PTP, indicating that HEMERA is an echoprotein directly translocated from plastids to the nucleus [73].

NCP (nuclear control of REP activity) [75] is a paralogue of RCB (regulator of chloroplast biogenesis) [76]. Both proteins are required for activation of plastid gene expression by phytochrome signalling during chloroplast development. They have both been detected to have the same molecular weights in plastids and the nucleus. The two proteins belong to those ON proteins with an eclipsed distribution. They were originally detected as plastid proteins, dubbed SVR4 (MRL7) and SVR4-like (MRL7-like) [77,78] (electronic supplementary material, table S1). SVR4 has been found to be an intrinsic component of highly purified nucleoids [79]. Both paralogues have an obvious impact on the architecture of nucleoids, as shown by DAPI staining of nucleoids in mutant plants [79]. Their impact on plastid gene expression hence could be at least partly caused by changes in compaction of nucleoids.

PAPs (PEP-associated proteins). Twelve PAPs have been identified to associate with PEP in plastids [66,69]. Six PAPs were also identified in the transcriptionally active chromosome and were dubbed pTAC proteins [67]. PAP1, 7, 8 and 12 are predicted to locate to the nucleus like PAP5, which is identical with pTAC12/HEMERA (see above). Co-expression analyses revealed a high degree of co-regulation in different tissues preceding the development of chloroplasts [80]. Interaction studies revealed that the PAPs form a large complex with PEP [81], the assembly of which seemingly depends on NCP and RBC [75,76].

Whirly1 is a major nucleoid-associated protein of chloroplasts [4,67,82]. In maize, Whirly1 has been shown to promote chloroplast development by its positive impact on plastid ribosome formation [83], as also obvious from the delayed development of the photosynthetic apparatus in RNAi-mediated Whirly1 knockdown plants of barley [84]. However, the protein has no effect on chloroplast development in *Arabidopsis*, where it lacks the PRAPP motif responsible for packaging of nucleoids [85] (table 1).

**Table 1.** Selected dually localized plant proteins playing roles in genome coordination. Localization and nucleic acid-binding motifs are indicated. The coordinative functions are proposed based on the data available. M, mitochondria; N, nucleus; P, plastids; PEP, plastid-encoded RNA polymerase.

protein	compartment	nucleic acid-binding motifs	(putative) coordinative function	references
HEMERA, pTAC12, PAP5	P, N	Glu-rich	coordination of PEP activity and photomorphogenesis	[70,73,74]
MFP1	P, N	coiled-coil	unknown	[86]
MSH1	P, M	FYE	link between organelle genome stability and epigenetics	[87,88]
NCP, MRL7-L, SVR4-L	P, N	Glu and Asp-rich	phytochrome control of PEP assembly and chloroplast development, ptNAP	[75,77–79]
OR	P, N	DnaJ-like zinc finger	development of carotenoid-accumulating plastids	[89]
PAP1	P, N	SAP	nuclear control of PEP and chloroplast development	[69,81]
PEND	P, N	leucine zipper bZIP	link between nucleoid architecture and photosynthesis-associated nuclear gene expression	[90–92]
PMN1	M, N	PPR, helix–turn–helix	link between mitochondrial translation and nuclear gene expression	[51]
RCB, MRL7, SVR4	P, N	Glu and Asp-rich	link between organelle genome stability and epigenetics	[76,77,79]
SWIB-4	P, M	SWIB	putative role in coordinated packaging of nucleoids and remodelling of chromatin	[82]
SWIB-6	M, P	SWIB	putative role in coordinated packaging of nucleoids	[82]
Whirly1	P, N	KGKAAL, PRAPP	plastid signalling linked to salicylic acid and abscisic acid-dependent nuclear gene expression	[93–96]
Whirly3	M, P	KGKAAL	role in coordination of organelle functionalities	M Zottini, K Krupinska 2019, unpublished data

All proteins of this group show high expression at early stages of development. However, the abundance of the proteins may differ considerably. For example, while the level of SRV4 (RCB) increases during chloroplast development in barley, the level of SVR4-like decreases [79]. Mutant analyses revealed that each of these proteins is required for chloroplast development. In conclusion, chloroplast development is controlled by at least seven PN proteins. Since all have the same molecular weight in plastids and the nucleus, they all seem to be relocated from plastids to the nucleus, as already experimentally demonstrated for Whirly1 and HEMERA [73,93] (§5 and figure 3).

#### (ii) Proteins associated with stress signalling

Chloroplasts are sensors of environmental change and are required for the production of major hormones adjusting plant metabolism to adverse environmental conditions [97]. During pathogen stress, high light intensity and exposure to UV, about 90% of salicylic acid (SA) was found to be produced in plastids, together with ROS as by-products of photosynthesis [98,99]. It is hence likely that stress-associated hormone signalling initiates in plastids and involves proteins that relocate to the nucleus [16].

ANAC102 (*Arabidopsis* NAC transcription factor 102) is a stress-associated transcription factor found to be located in plastids when C-terminally fused with GFP. By contrast, a fusion with GFP at the N-terminus was identified in the nucleus [100]. Expression of *ANAC102* is responsive to hydrogen peroxide [100], enhanced during excess light and upon treatment with the carotenoid catabolite β-cyclocitral, which is a component of plastid signalling [101]. The *anac102* mutant is impaired in responses to β-cyclocitral, indicating that ANAC102 is a master regulator in the establishment of tolerance towards photooxidative stress downstream of β-cyclocitral-mediated plastid signalling [101].

NRIP1 (N receptor interacting protein 1) is a defence protein shown to be translocated from the chloroplast to the nucleus by fusion with the cerulean fluorescent protein (§4b). The translocation can be induced by treatment of plants with the pathogen effector molecular p50 [102]. Sequence analysis revealed that the protein is homologous to the chloroplast-located and senescence-associated AtSEN1 protein [103], which has a function in the biosynthesis of molybdenum cofactors required for enzymes such as xanthine dehydrogenase that are involved in the regulation of ROS [104].

Orange (OR) [89,105] is a dually targeted chaperone with a DnaJ-like zinc finger domain that plays a role in the transition from non-pigmented plastids into carotenoid-accumulating chromoplasts [89,106]. Overexpression in plants was shown to enhance carotenoid accumulation and tolerance to abiotic stress [107,108]. OR usually localizes in plastids, where it interacts with phytoene synthase [109]. In etiolated cotyledons of *Arabidopsis* [105], it has been also found in the nucleus, where it has a higher molecular weight owing to the presence of the PTP, indicating distribution by dual targeting [105,110].

RAF2 (Rubisco assembly factor 2) under normal conditions is located in plastids, where it aids in the assembly of Rubisco [111]. In the nucleus, the protein functions as a cofactor in regulation of defence-related genes. The intracellular distribution of NbRAF2, which in the nucleus has the molecular weight of the mature plastid protein, is affected by interaction with viral proteins [111]. Interaction with potato leafroll virus Po^PL^ decreased the nuclear pool of NbRAF2 and thereby might facilitate virus infection.

Sigma factor binding protein 1 (SIB1) was first identified as an interaction partner of the plastidic sigma factor 1 of PEP [112] and was shown to activate transcription of *WRKY33* during the plant defence response [113]. Recently, it has been shown that the dual targeting of SIB1 to chloroplasts and the nucleus in response to SA leads to a simultaneous change in transcription of photosynthesis-associated genes in the two compartments [114]. While the photosynthesis-associated plastid genes (PhAPGs) are down-regulated, the photosynthesis-associated nuclear genes (PhANGs) are up-regulated. Owing to the induced imbalance of the stoichiometry in photosystem II, singlet oxygen is produced, which is known to participate in retrograde signalling to induce a cell death programme [115].

Whirly1 is a multifunctional protein that before its detection in chloroplasts was implicated in SA signalling [94,95,98]. Its binding to pathogen response promoters in the nucleus was shown to depend on SA. Accordingly, an *Arabidopsis* *why1* mutant has a reduced sensitivity towards SA [96]. It has been proposed that an inactive pool of Whirly1 is activated by SA [94]. Likely, this inactive pool is the chloroplast pool of Whirly1.

#### (iii) Coordination of plastid division and cell cycle

CDC10 Target 1 (CDT1) is a kinase involved in cell cycle regulation. In the nucleus, it interacts with DNA polymerase ɛ and functions in replication and response to DNA stress/maintenance of genome integrity [116]. CDT1-*RNAi* plants show endogenous DNA stress and are more tolerant to DNA-damage-inducing agents owing to constitutive expression of genes encoding DNA repair proteins [116]. CDT1a, but not CTD1b, has a functioning PTP [117] and was furthermore shown to function in plastid division. This could indicate that CDT1a plays a role in the coordination of plastid division and cell cycle [116].

ATXR5 (*Arabidopsis* trithorax-related 5) is an SET domain protein involved in the regulation of replication and DNA repair [118]. Its localization in the organelle has been proposed to provide a means to keep it out of the nucleus, where it is active.

#### (iv) DNA association of PN proteins

A couple of the PN proteins have DNA-associated functions and hence are candidates for genome coordination by affecting on one hand shaping and organization of nucleoids and transcriptional activity in the organelle and on the other hand nuclear gene expression (table 1). Their binding to DNA is mediated by typical eukaryotic DNA-binding motifs such as the coiled-coil motif (MFP1), a zinc finger (OR), an SAP motif (PAP1), a leucine zipper (PEND) or the SWIB domain. The nucleoid-associated proteins SVR4 and SVR4-like, identical with RCB and NCP respectively, as well as HEMERA are enriched in negatively charged amino acids, which is a characteristic feature of chaperones assisting in assembly and maintenance of DNA–protein complexes [3,79,119]. SWIB-4 is the only PN member of the group of small SWIB (SWI/SNF complex B) domain-containing proteins identified in *Arabidopsis* [82]. The protein was shown to induce compaction and condensation of nucleoids and to functionally complement a mutant of *Escherichia coli* lacking the histone-like nucleoid structuring protein H-NS [82] and likely has a chromatin-associated function in the nucleus. Whirly1 has been shown to bind as a single-stranded DNA-binding protein to a specific *cis*-element in promoters of pathogenesis related (PR) genes [95,120]. By contrast, in plastids Whirly1 binds unspecifically to DNA [83] and promotes compaction of nucleoids [120] caused by the PRAPP motif, which is shared by the Whirly1 proteins in monocots such as barley and maize [85].

Regulated changes in the distribution of such genome-coordinating proteins likely present a strategy to shift patterns of gene expression in the organelles and the nucleus, as clearly demonstrated for SIB1 in a stress situation [114].

### (c) DNA-associated proteins dually targeted to mitochondria and plastids

Owing to ambiguous OTP sequences, in plant cells numerous proteins were found to be dually targeted to both DNA-containing organelles [121,122]. In the framework of this review, we selected only those with DNA-associated functions being potentially involved in genome communication (table 1).

#### (i) Proteins involved in DNA repair

MutS homologue 1 (MSH1). In plant mitochondria recombination between mtDNA molecules is frequent and common [123], requiring control by a ‘recombination surveillance’ machinery. MSH1 [124] is a key component of this machinery and a disturbance of the machinery leads to an increase in the recombination rate of mtDNA [125]. *msh1* mutants show leaf variegation arising by incomplete development or premature degeneration of plastids. Plastid genome rearrangements in white sectors of mutant leaves revealed that MSH1 functions in both organelles [124]. Disturbances in the organelles give rise to retrograde signalling and have an impact on nuclear gene expression and consequently growth, developmental processes and abiotic stress responses. Retrograde signalling has been proposed to be mediated by ROS and to interact with hormone signalling [126]. The magnitude of the complex and partially environment-dependent phenotypes of *msh1* mutants involves heritable changes in small RNAs and chromatin organization that increase according to the mutant generation [87,126].

RECG is a plant-specific orthologue of the bacterial DNA helicase RECG. RECG localizes to both chloroplast and mitochondrial nucleoids and has multiple roles in mtDNA repair. In particular, it is required for recombination-dependent repair and for suppression of ectopic recombination in mitochondria, most likely because of its role in the recovery of stalled replication forks [127]. Knockout of RECG causes growth defects and abnormal ultrastructure of chloroplasts and mitochondria and leads to instability of the organelle genomes as a result of recombination [128]. The dual localization of RECG indicates that the mechanisms underlying the suppression of aberrant recombination are shared by plastids and mitochondria.

#### (ii) Replication enzymes

Plant organelle polymerase (POP) [129]. Plastid and mitochondria share one or two enzymes with homology to bacterial DNA polymerase I. In *A. thaliana,* two POP genes are differentially expressed in different tissues [130]. While POLIA seems to be exclusively involved in replication, POLIB might also function in the repair of DNA [131].

Twinkle is a homologue of a T7 phage protein functioning during replication as a DNA helicase and primase [132]. In fusion with GFP, TWINKLE was shown to localize to both mitochondria and chloroplasts [133].

Topoisomerases prevent and correct topological problems when DNA becomes overwound at the replication fork. Both type II (gyrase A) and type A enzymes have been shown to localize to both organelles in *Arabidopsis* (for reviews, see [134]).

#### (iii) Proteins involved in gene expression and nucleoid architecture

RNA polymerase of the phage-type (RPOTmp) is a third nuclear-encoded organelle RNA polymerase targeted to both organelles. It is only found in some plants, including *A. thaliana* and *Nicotiana tabacum* [135]. While in mitochondria RPOTmp plays a major role in the regulation of gene expression, in chloroplasts it seems to be important only at early stages of plant development [136].

SWIB-6 [82] belongs to the SWIB domain-containing proteins which are subunits of nuclear ATP-dependent chromatin-remodelling complexes of the SWI/SNF type (§3b). SWIB-6 has been demonstrated to localize in both chloroplasts and mitochondria as a nucleoid protein and could be involved in the architecture of nucleoids, acting in a similar way to the nuclear SWIB complex [137] and SWIB-5 in mitochondria, which interacts with other SWIB proteins in the organelle [138].

Whirly3 is a homologous protein to Whirly1 that is only present in *A. thaliana* and other species of the family Brassicaceae. Although predicted to be a plastid protein, it has recently been shown to be dually imported into chloroplast and mitochondria by an organelle protein transporter (B Bennewitz, RB Klösgen, K Krupinska, M Zottini 2019, unpublished data). Accordingly, Whirly3 can replace mitochondrial Whirly2 in a *why2* knockout mutant at certain stages of development.

## 4. Mechanisms of dual targeting and relocation

### (a) Dual targeting to organelles and nucleus

The above sections demonstrate that the dual localization of proteins in the nucleus and endosymbiotic organelles are rather a common phenomenon. Various alternative mechanisms have been proposed or partially revealed to underlie the dual distribution of a single gene product between organelles and the nucleus, as summarized by Krause & Krupinska [17]. In general terms, these mechanisms can be classified into two major categories: dual targeting to organelles and nucleus (§4a) or relocalization of a protein from one organelle to nucleus (§4b). In the first category, two principal strategies involve the formation of multiple proteins from different transcription or translation start sites resulting in proteins with different targeting information or a post-translational modification of a single protein.

An interesting example of dual targeting is displayed by a bZIP transcription factor named activated transcription factor associated with stress 1 (ATFS-1). The distribution of ATFS-1, which harbours an NLS besides an MTP sequence, is dependent on the mitochondrial import efficiency, which in turn depends on the functioning of the organelle [140]. In *Caenorhabditis elegans*, ATFS-1 regulates a signalling mechanism named mitochondrial unfolded protein response (UPR^mt^). The UPR^mt^ is triggered by several situations affecting the functionality of mitochondria, such as disturbed OXPHOS and ROS, and induces a block in the mitochondrial import machinery [141]. Whereas under control conditions ATFS-1 is efficiently imported in mitochondria, upon UPR^mt^ induction, the mitochondrial pool of ATFS-1 is stabilized, binds to mtDNA and down-regulates the level and turnover of mitochondrial mRNAs [25]. Owing to impaired mitochondrial protein import, ATFS-1 is targeted to the nucleus, where it down-regulates the expression of genes involved in OXPHOS [25].

Events like alternative splicing at the N-terminal or multiple transcription or translation start sites might affect the hierarchy for preferential targeting of a protein to organelles and nucleus. For example, the longer version of *Arabidopsis* DNA ligase 1 (§3a), encoded from the first AUG, harbours both an MTP sequence and an NLS and is exclusively targeted to mitochondria, confirming the dominance of the mitochondrial importing machinery over nuclear importation. A second downstream AUG generates an alternative translation initiation, dependent on secondary RNA structure, and leads to a shorter protein, which only harbours the NLS and is specifically targeted to the nucleus. A similar scenario applies to TRXO and PNM1 (§3a), which both contain the NLS in the C-terminal part of the sequence.

An overlap of NLS and OTP can provide different scenarios of controlled intracellular distribution. SIB1 (§3b) was identified in chloroplasts and the nucleus, harbouring a 54 amino acid PTP with an internal NLS [114]. Upon import into plastids, the PTP including the NLS is cleaved, trapping the protein in the organelle. Both pools increase upon SA treatment and lead to simultaneous changes in gene expression in both compartments [114] (§3b).

### (b) Translocation of organelle proteins to the nucleus

The distribution of echoproteins having the same molecular weight in organelles and nucleus cannot be explained by dual targeting from the cytoplasm. Although not fully understood, different mechanisms of redistribution between organelles and nucleus are conceivable.

**Figure 2.**
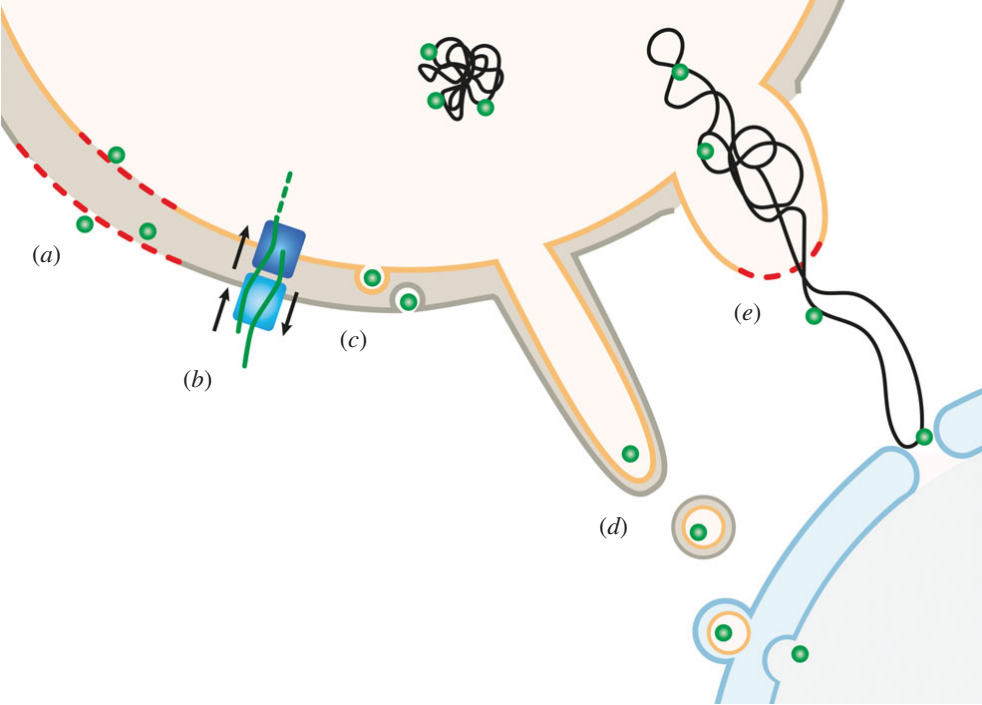
Schematic of putative mechanisms of protein release from the organelles and translocation into the nucleus, modified according to Krause & Krupinska [17]: release of proteins from damaged organelles (*a*); retranslocation via the translocon complexes (TOM/TIM, TOC/TIC) after processing (*b*); vesicle-mediated efflux of proteins (*c*); stromule tip shedding and fusion of double-bounded vesicles with the nuclear envelope (*d*); stress-induced escape of proteins bound to organelle DNA (*e*).

Fumarase, the first described echoprotein, is relocated to the nucleus after processing in the organelle [142]. Thereby the protein is released from mitochondria via TOM40 as an escape gate—a mechanism proposed to control also the mitochondrial pools of numerous other proteins [143]. However, large protein complexes such as PDC, which in human cells has been found to be translocated upon stress from mitochondria to the nucleus, indicate the existence of at least one other relocation mechanism [29]. Several possibilities for such a translocation mechanism have been proposed: release via mitochondria-derived vesicles [144], retrotranslocation of PDC components following cleavage of the N-terminal MTP with subsequent formation of the complex in the cytosol and its translocation to the nucleus, or most simply and most likely the leakage from damaged mitochondria (figure 2) [22].

#### (i) Involvement of protrusions from the organelles in protein transfer

Under certain conditions organelles form protrusions which have been named stromules in the case of plastids [145] or matrixules in the case of mitochondria [146]. In chloroplasts, these structures occur at higher frequency in stress situations associated with the formation of ROS and SA [145]. It is still a matter of debate whether these structures are involved in transfer of proteins from organelles to the nucleus or a sign of chloroplast malfunction. The best evidence for their involvement in protein transfer comes from the impressive study of the pathogen-induced relocation of the defence protein NRIP1 from plastids to the nucleus [102] (§3b).

In plastids, NRIP1 recognizes the effector protein p50 helicase from TMV and translocates with it to the cytosol and nucleus. The transfer could occur either by shedding of vesicles from stromules or simply by damage of membranes as a result of pathogen-induced stress coinciding with the production of ROS and SA (figure 2). This latter scenario was described for stroma-targeted GFP in conditions of biotic and abiotic stress that provoke an increase in ROS inside chloroplasts [147,148].

#### (ii) Translocation of organelle DNA to the nucleus

It is striking that most of the ON proteins bind to DNA, enabling them to functions as transcription factors, chromatin-remodelling factors or nucleoid architectural proteins (figure 1 and table 1). Considering that organelle DNA is continuously transferred to the nucleus, leading to a considerable fraction of organelle DNA in the nucleus [149], it seems possible that nucleoid proteins are translocated to the nucleus while attached to organelle DNA. DNA transfer from organelles has been determined with transplastomic plants containing a nuclear-selectable marker gene [150,151]. Its frequency was shown to be highest during degradation of organelle DNA during male gametogenesis in plants with maternal inheritance of organelles [152]. It is possible that DNA is translocated to the nucleus in the form of nucleoprotein complexes such as those formed by viral movement proteins [153] or simply by release of fragmented organelle DNA from damaged chloroplasts/mitochondria which after random arrival in the nucleus might get integrated into the genome [153].

The mechanism whereby mtDNA is released into the cytosol of mammalian cells upon apoptotic stimuli has recently been elucidated [154,155]. After permeabilization of the inner mitochondrial membrane, leading to release of apoptotic factors such as cytochrome *c*, a gradual widening of the pores in the outer membrane was observed to induce a release of mtDNA to the cytosol, as monitored by super-resolution imaging [154].

All ON proteins so far described in plants have been detected in organelles and in the nucleus, but not in the cytosol. Although it seems obvious to expect such a protein in the cytosol, so far there is no evidence for a cytosolic localization [16]. It is hence likely that translocation from organelles to the nucleus requires close contact between the two compartments. Indeed it has been shown that chloroplasts are tightly associated with the nucleus during high light exposure [156], a situation known to be associated with chloroplastic production of SA [99] and potential damage of organelle membrane structure by SA [157]. Indeed, damage of membranes seemingly is a prerequisite of stress-induced plastid signalling that is experimentally induced, often by treatments with the herbicide norflurazon [158].

Physical interactions of organelles with the nucleus might be an important prerequisite for the transfer of DNA to the nucleus [159]. During certain stages of plant development, mitochondria and chloroplasts have been observed even inside the nucleus by electron microscopy [160,161]. Such events are, however, rare and might only occur when the nuclear envelope is disintegrating during cell division.

## 5. Methods suited for analyses of organelle–nucleus translocation

### (a) Strategies used for analysis of the dual localization of selected proteins

In this review, the redistribution of proteins between endosymbiotic organelles and nucleus through various mechanistic models that are in line with the hypothesis of an initial organelle importation event and a subsequent translocation to the nucleus has been presented. These mechanisms of translocation fit either the idea of a link between the function of the dual-localized protein in each of its locations or/and a transduction of information from one compartment to the other to trigger a specific response. Several techniques and experimental approaches have been used to demonstrate the dual localization of a protein and/or its relocalization between endosymbiotic organelles and the nucleus.

**Figure 3.**
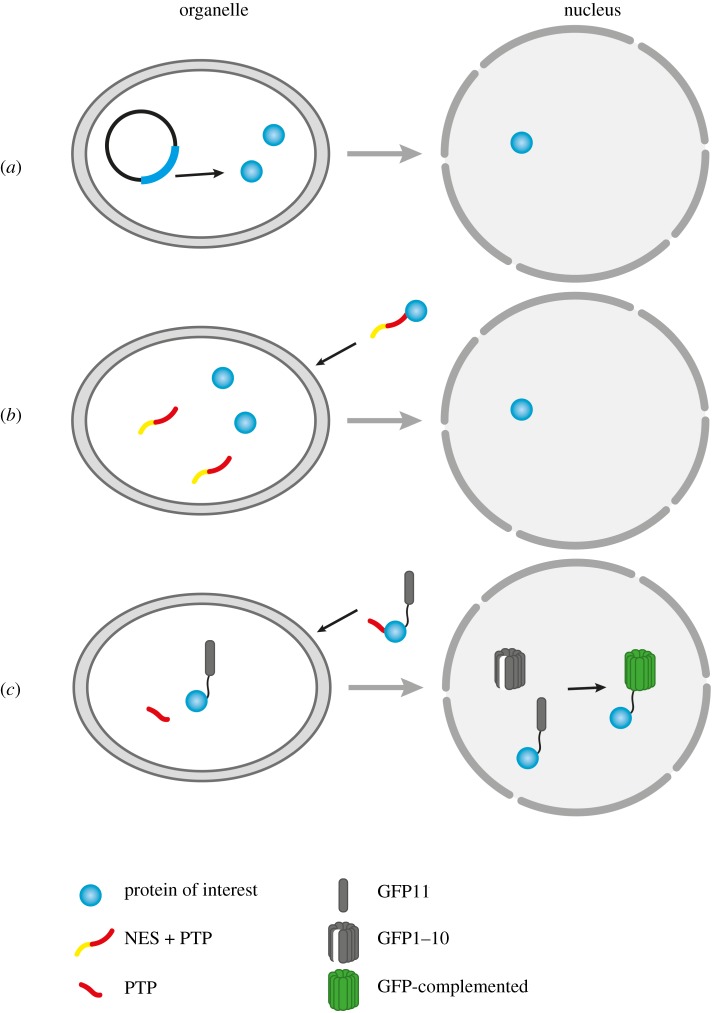
Methods allowing study of the relocation of proteins from organelles to the nucleus. Relocation of a recombinant tagged protein synthesized in transplastomic plants such as Whirly1 : HA [96] (*a*), targeting of a protein such as NRIP having a PTP and an NES [102] (*b*), self-assembly of split GFP in the nucleus, whereby the protein of interest is fused to GFP11 [162] (*c*).

One of the initial approaches to confirm a real organelle to nucleus translocation in human cells was the use of cycloheximide (CHX), an inhibitor of cytoplasmic translation. CHX treatment prevented de novo synthesis of e.g. the subunit E-1 of PDC, confirming that the simultaneous increase of the nuclear PDC-E1 level and the decrease of mitochondrial pool were consequences of a relocation from mitochondria [29]. Other strategies employed a combination of microscopic and biochemical techniques to reveal translocation processes. A straightforward and conclusive strategy to show translocation from plastids to the nucleus is the production of transplastomic plants and the subsequent detection of the protein in the nucleus (figure 3*a*). With transplastomic tobacco plants expressing a sequence encoding an HA-tagged version of AtWhirly1 lacking the PTP in the plastid genome [93], the protein was shown to relocate to nuclei, as demonstrated by immunofluorescence and immunogold microscopy using an antibody directed towards the tag [93]. The abundant nuclear localization of chloroplast-derived tagged Whirly1 coincided with enhanced expression of PR genes (§3b). This strategy is, however, rather time-consuming and most successful transformations have been done with tobacco plants [163].

Another elegant approach to demonstrate the relocation from plastids to the nucleus was reported for the defence protein NRIP1. A construct encoding an NES preceding the PTP of NRIP1 was fused with the cerulean fluorescent protein [102]. The nuclear localization was only detectable when the recombinant protein lost the NES as a result of processing inside chloroplasts (figure 3*b*). When NRIP1 was fused with the NES without the PTP, fluorescence was excluded from the nucleus and accumulated in the cytosol [102].

A way to minimize the impact of bulky fluorescence tags on folding features of the fusion proteins used in localization experiments of ON proteins is the usage of self-assembling split-fluorescent proteins [162,164]. The rationale behind this technique is the fusion of the candidate protein to a small part of the GFP protein (GFP11), which is targeted to organelles and after translocation from organelles combines with the larger portion of GFPs (GFP1–10) that is already present in the nucleus (figure 3*c*).

Conclusive results on the translocation of a protein from organelles to the nucleus can be also provided by hemicomplementation of mutants. Such a genetic approach has been undertaken in the case of HEMERA, which functions in phytochrome signalling as well as in plastid gene expression and chloroplast development (§3b). The complementation of the *hmr-5* mutant with the mature HEMERA fused to the PTP of the small subunit of Rubisco (ribulose-1,5-bisphosphate carboxylase/oxygenase) was able to fully restore the functions of HEMERA in chloroplasts on one hand and phytochrome signalling on the other hand, while complementation with a construct lacking the PTP failed to rescue these functions [73]. The results of this complementation approach are in accordance with the idea that HEMERA like Whirly1 is first targeted to plastids, where it is processed to the mature form and then relocated to the nucleus [73]. In the case of Whirly1, this approach could not be undertaken owing to the lack of an NLS [96]. A fusion of the full-length Whirly1 with an NES sequence excluding the protein from the nucleus was not successful (N Grabe, K Krause 2010, unpublished results).

Super-resolution fluorescence microscopy provides the promising prospect of directly tracking translocation of proteins from organelles to the nucleus. This technique has been recently employed to demonstrate the release of mtDNA from mitochondria [154]. It is likely that this technology can also trace a putative simultaneous release of DNA and DNA-binding proteins from the organelles. State-of-the-art microscopic techniques with augmented resolution have been tested for life-cell imaging in plants, which entails specific challenges such as high light scattering [165,166]. Furthermore, photo-convertible fluorescent proteins such as Dendra2 are emerging as useful tools in tracing a fraction of the whole protein cellular population on its way from an organelle to the nucleus [167,168].

### (b) Best practices for demonstrating dual location and relocation of proteins

The most obvious approaches to study dual localization rely on *in vitro* methods such as transient transformation of protoplasts, agro-infiltration of leaves or biolistic transformation of different tissues with constructs encoding fusion proteins with a fluorescent tag. In addition, proteins with predicted localization in organelles are tested by in-organelle protein import assays. These approaches are usually performed with standard systems and model organisms, such as mesophyll protoplasts prepared from fully developed leaves, epidermal cells from onions and chloroplasts from pea leaves. The results of these approaches might be erroneous and conflicting in the case of proteins with specific spatio-temporal regulation. Indeed, most ON proteins, e.g. DHFR-TS (§3a) and those PN proteins required for chloroplast development (§3b), show tissue- and development-dependent changes in expression and accumulation. Therefore, the *in vitro* studies have to be complemented by immunological methods such as immunogold labelling and immunoblot analysis of subcellular fractions. A combination of techniques might be also applied to studies of hemicomplementation of mutants to support the phenotypic observations.

Results obtained by biochemical and microscopic investigations can be further fostered by information derived from the curated database SUBA4, which offers an integrated collection of published information about protein subcellular localization based on large-scale subcellular proteomics, fluorescent protein visualization, protein–protein interaction and prediction programmes [169]. Enhanced sensitivity of mass spectrometry-based proteomics combined with improved subcellular fractionation will provide more information on the subcellular distribution of proteins [170].

Taken together, there is no obvious unique method to determine reliably the subcellular distribution of a protein in eukaryotic cells. To avoid erroneous results or misinterpretations, studies on protein localization should combine complementary strategies, ideally involving the phenotypic and functional characterization of mutants complemented with compartment-specific sequences.

## 6. Conclusion

Dual targeting or dual localization of proteins in eukaryotic cells has eventually been accepted as an important phenomenon linked with multi-functionalization. The knowledge on ON proteins presented in this review indicates that dual localization is an obvious strategy to tighten and coordinate genome-related functions in organelles and the nucleus.

Organelles are critical integrators of both internal and external cues, and activities in the organelles need to be tightly coordinated with nuclear activities to enable plant development and stress signalling. It is tempting to speculate that changes in the distribution of those ON proteins binding to DNA between organelles on one hand and the nucleus on the other hand might be an efficient and orchestrated way to adjust gene expression in the two compartments by changes in nucleoid architecture and nuclear chromatin remodelling. Candidate proteins for such a coordinated control of organelle and nuclear genomes are, e.g., the SWIB and Whirly proteins. While in plants studies on the variability of nucleoid architecture and its significance for plant growth and stress resistance are still in their infancy, in humans induced changes in the architecture of nucleoids by mtDNA-binding proteins considered as ‘mito-epigenetics’ have gained increasing attention with regard to their impact on health [171].

Dual localization of proteins in eukaryotic cells has been proposed to have an evolutionary advantage [172]. In accordance with their multi-functionality featuring combinations of diverse motifs in one polypeptide chain, dual-located proteins are evolutionarily more conserved than proteins exclusively found in one compartment [173]. In higher plants, the evolutionary advantage of dual-located plastid proteins might be linked to the multitude of organelles in one cell. In contrast to algae, which usually have one or very few chloroplasts per cell, photosynthetic tissues of higher plants possess 50 to hundreds of chloroplasts. It would be sufficient to release a protein from one or a few plastids from the multitude of plastids within one cell to rapidly change gene expression in the nucleus under conditions of stress [17]. Although systematic research linking organelle number per cell, retrograde signalling and plant stress resistance remains to be done, it is obvious that multi-functionalization of proteins such as Whirly1 is a recent evolutionary strategy to increase the adaptability and robustness of plants, enabling them to survive in an ever-changing environment [174].

## Supplementary Material

Supplementary Table 1

## Data accessibility

This article has no additional data.
