# Supplementary material for: Genome communication in plants mediated by organelle–n­ucleus-located proteins
Source: Philos Trans R Soc Lond B Biol Sci. 2020 May 4;375(1801):20190397. doi: 10.1098/rstb.2019.0397 (PMC7209962; doi:10.1098/rstb.2019.0397)
Supplement: Supplementary Table 1 [file rstb20190397supp1.pdf]

Karin Krupinska, Nicolás Blanco, Svenja Oetke, Michela Zottini

Genome communication in plants mediated by organelle-nucleus located proteins

Philosophical Transaction B

Supplementary Table 1. List of organelle-nucleus located plant proteins (ON)

protein name(s)	localization method(s)	function in organelles/plastids	function in the nucleus	relocation or dual targeting	references
ADT5	P: CFP<br>N: CFP	phenylalanine biosynthesis	unknown	possible relocation	Bross et al. 2017
ANAC102	P: GFP<br>N: pred (NLS)	component of β-cyclocitral-mediated retrograde signalling	NAC transcription factor	not investigated	D'Alessandro et al. 2008, Inze et al. 2012
ANNEXIN 5	P: YFP<br>N: YFP	interaction with RABE1b, a putative GTPase	unknown	not investigated	Lichocka et al. 2018
ATXR5	P: GFP<br>N: GFP	unknown	control of cell cycle and DNA replication<br>H3K27 methyltransferase	not investigated	Jacob et al. 2009, 2010, 2014, Raynaud et al. 2006
CDT1	P: GFP<br>N: GFP	plastid division (interaction with ARC6)	DNA replication (interaction with DNA polymerase ε)	not investigated	Domenichini et al. 2012, Raynaud et al. 2005
cp29B (P)<br>SEBF (N)	P: imp, prot<br>N: biochem	RNA-binding protein	transcriptional repressor	not investigated	Boyle + Brisson 2001, Ohta et al. 1995, Ruwe et al. 2011
cp31A (P)<br>STEP1 (N)	P: imp, prot<br>N: GFP	RNA-binding protein; cold stress tolerance by influencing chloroplast RNA processing	telomere-binding protein	not investigated	Kupsch et al. 2012,<br>Kwon + Chung 2004, Ohta et al. 1995, Ruwe et al. 2011, Yoo et al. 2010

DHFR-TS	P: gold, blot, GFP<br>M: act, GFP<br>N: gold, GFP	dihydrofolate reductase-thymidylate synthase	dihydrofolate reductase-thymidylate synthase	not investigated	Luo et al. 1997
ERF34	P: GFP<br>N: GFP	unknown	transcription factor with AP2 DNA binding motif	not investigated	Saelim et al. 2018, Schwacke et al. 2007
HEMERA (N) pTAC12/PAP5 (P)	P: prot, blot, fluo<br>N: blot, fluo	nucleoid associated protein; PEP associated protein (PAP)	transcriptional activator in phytochrome signalling	relocation	Chen et al. 2010, Galvao et al. 2012, Pfalz et al. 2006, Pfalz et al. 2015, Qiu et al. 2015, Steiner et al. 2011
IPT3	P: GFP<br>N: GFP	cytokinin biosynthesis	unknown	not investigated	Galichet et al. 2008
LEM1	P: GFP<br>N: GFP	putative component of ribosomes (PRPS9)	required for embryogenesis	not investigated	Ma + Dooner 2004
LIGASE1	P: GFP<br>M: GFP	DNA replication and excision repair pathways	DNA replication and excision repair pathways	dual targeting	Sunderland 2006
MFP1	P: gold, fluo GFP, blot<br>N: fluo, blot	nucleoid associated protein with a function in thylakoid association and starch granule formation	matrix attachment region binding	not investigated	Jeong et al. 2003, Meier et al. 1996, Samaniego et al. 2006, Seung et al. 2018,
NCP, MRL7, SVR4	P: CFP, blot<br>N: CFP, blot	nucleoid associated protein promoting compaction; assembly factor of PEP	degradation of PIF1 and PIF3	possible relocation	Powikrowska et al. 2014, Qiao et al. 2011, Yang et al. 2019
NRIP1	P: cer<br>N: cer	rhodanese sulfur transferase; immune receptor recognition; plastid function unknown	mediates innate immune receptor recognition by a viral effector	relocation	Caplan et al. 2015
NtWIN4	P: blot, GFP<br>N: GFP	unknown	induction of hypersensitive cell death; transcriptional repressor	dual targeting	Kodama et al. 2006
OPENER	N: YFP<br>M: YFP	mitochondria dynamics	involved in nuclear envelope and nucleoli stability	relocation	Wang et al. 2019
OR	P: GFP, blot, BiFC<br>N: GFP, blot	chromoplast development	unknown	dual targeting	Sun et al. 2016, Zhou et al. 2015

PAP1,7,8,9,12	P: prot<br>N: pred	chloroplast development	unknown	unknown	Pfannschmidt et al. 2015, Steiner et al. 2011
PEND (P)<br>GSBF1 (N)	P: blot, imp, GFP<br>N: GFP	nucleoid associated protein	transcription factor	possible relocation	Sato et al. 1998, Terasawa and Sato 2009, Waldmüller et al. 1996
PBH3	N:YFP, blot<br>M: YFP	regulates mitochondria functionality	regulates DNA replication and genome stability	relocation	Huang et al. 2019
PMN1	M: GFP/YFP<br>N: GFP/YFP	associated with polysomes; role in translation	regulates transcription	dual targeting	Hammani et al. 2011
RAF2	P: RFP<br>N: RFP	Rubisco assembly chaperone	regulator of immune responses	relocation	Sun et al. 2018
RCB, MRL7-like, SVR4-like	P: CFP, blot<br>N: CFP, blot	promotes compaction of nucleoids; assembly factor of PEP	promotes localization of phytochromes to photobodies	possible relocation	Powikrowska et al. 2014, Qiao et al. 2011, Yoo et al. 2019
SIB1	P: GFP<br>N: GFP, BiFC	proteins binding to sigma factor 1 of PEP	SA-dependent transcriptional regulator; modulation of defense reactions; interaction with WRKY33 and 57	not investigated	Jiang et al. 2016, Lai et al. 2011, Morikawa et al. 2002, Lv et al. 2019
SRT1/2	N: blot<br>M: blot, GFP	regulates mitochondrial activity by protein deacetylation	repressing ethylene dependent gene repression through histone deacetylase activity	relocation not investigated	König et al. 2014
SWIB-4	P: GFP, prot<br>N: GFP	DNA packaging	unknown	dual targeting	Melonek et al. 2012
TRXO	N: gold, GFP<br>M: gold, GFP	activation of alternative oxidase	putative protection of the genome against oxidation and control of transcription of noncoding DNA	dual targeting	Marti et al. 2009
WHIRLY1	P: blot, GFP, imp, gold<br>N: blot, gold	DNA + RNA binding; DNA maintenance; nucleoid architectural protein	transcriptional activator of PR genes; repressor of the HvS40 gene; telomere-binding protein	relocation	Desveaux et al. 2000, 2004, Grabowski et al. 2008, Krause et al. 2005, Krupinska et al. 2014 a/b, Yoo et al. 2007

ABBREVIATIONS USED FOR PROTEINS: ADT5 = arogenate dehydratase 5; ANAC102 = Arabidopsis NAC transcription factor 102; ATXR5 = Arabidopsis trithorax-related 5; CDT1 = CDC10 target 1; cp29B/SEBF = silencing element binding factor/chloroplast RNA-binding protein 29B/; cp31A/STEP1 = chloroplast RNA-binding protein 31A/single-stranded telomere-binding protein 1; DHFR = dihydrofolate reductase; ERF34 = ethylene response factor; IPT3 = adenosine phosphate-isopentenyltransferases; LEM1 = lethal embryo 1; MFP1 = matrix attachment region-binding filament-like protein 1; NCP/MRL7/SVR4 = nuclear control of PEP activity/mesophyll-cell RNAi Library line 7/suppressor of variegation 4; NRIP1 = N receptor interacting protein 1, NtWIN4 = *Nicotiana tabacum* wound-induced clone 4; OR = ORANGE; PAP = PEP-associated protein; PBH3 = prohibitin 3; PEND/GSBF1 = plastid envelope DNA binding/GS-box binding factor 1; PEP =plastid-encoded RNA polymerase; PIF = phytochrome interacting factor; PNM1 = PPR protein localized to the nucleus and mitochondria 1; pTAC12 = plastid transcriptionally active chromosome 12; RAF2 = Rubisco assembly factor 2; RCB/MRL7-like/SVR4-like = regulator of chloroplast biogenesis/mesophyll-cell RNAi Library line 7 like/suppressor of variegation 4 like; SIB1 = sigma factor binding protein 1; SRT1/2 = Sirtuin 1/2; SWIB-4 = SWIB-domain containing protein 4; TRXO = thioredoxin.

GENERAL ABBREVIATIONS: P = plastids, M = mitochondria, N = nucleus, GFP, CFP, RFP, YFP = fluorescent fusion proteins, fluo = immunofluorescence, blot = immunoblot analysis, gold = immunogold labelling, act = enzyme activity, imp = in vitro import, prot = proteomics, biochem = biochemical fractionation, cer = cerulean, BiFC = bimolecular fluorescence complementation, pred = prediction

REFERENCES

Boyle B, Brisson N (2001) Repression of the defense gene PR-10a by the single-stranded DNA binding protein SEBF. Plant Cell 13: 2525–2537

Bross CD, Howes TR, Rad SA, Kljakic O, Kohalmi SE (2017) Subcellular localization of Arabidopsis arogenate dehydratases suggests novel and non-enzymatic roles. J Exp Bot 68: 1425-1440

Caplan JL, Kumar AS, Park E, Padmanabhan MS, Hoban K, Modla S, Czymmek K, Dinesh-Kumar SP (2015) Chloroplast Stromules Function during Innate Immunity. Dev Cell 34: 45-57

Chen M, Galvao RM, Li MN, Burger B, Bugea J, Bolado J, Chory J (2010) Arabidopsis HEMERA/pTAC12 Initiates Photomorphogenesis by Phytochromes. Cell 141: 1230-U1237

Galichet A, Hoyerova K, Kamínek M, Gruissem W (2008) Farnesylation directs AtIPT3 subcellular localization and modulates cytokinin biosynthesis in Arabidopsis. Plant Physiol 146: 1155–1164

D'Alessandro S, Ksas B, Havaux M (2018) Decoding beta-cyclocitral-mediated retrograde signaling reveals the role of a detoxification response in plant tolerance to photooxidative stress. Plant Cell 30: 2495-2511

Desveaux D, Després C, Joyeux A, Subramaniam R, Brisson, N (2000) PBF-2 is a novel single-stranded DNA binding factor implicated in PR-10a gene activation in potato. Plant Cell 12: 1477-1489

Desveaux D, Subramaniam R, Deprés C, Mess J-N, Lévesque C, Fobert PR, Dangl J, Brisson N (2004) A “Whirly” transcription factor is required for salicylic acid-dependent disease resistance in Arabidopis. Dev Cell 6: 229-240

Domenichini S, Benhamed M, De Jaeger G, Van De Slijke E, Blanchet S, Bourge M, De Veylder L, Bergounioux C, Raynaud C (2012) Evidence for a role of Arabidopsis CDT1 proteins in gametophyte development and maintenance of genome integrity. Plant Cell 24: 2779-2791

Galvao RM, Li MN, Kothadia SM, Haskel JD, Decker PV, Van Buskirk EK, Chen M (2012) Photoactivated phytochromes interact with HEMERA and promote its accumulation to establish photomorphogenesis in Arabidopsis. Gene Dev 26: 1851-1863

Gorelova V, De Lepeleire J, Van Daele J, Pluim D, Meï C, Cuypers A, Leroux O, Rébeillé F, Schellens JHM, Blancquaert D, Stove CP, Van Der Straeten D (2017) Dihydrofolate reductase/thymidylate synthase fine-tunes the folate status and controls redox homeostasis in plants. Plant Cell 29: 2831-2853

Grabowski E, Miao Y, Mulisch M, Krupinska, K (2008) Single-stranded DNA-binding protein Whirly1 in barley leaves is located in plastids and the nucleus of the same cell. Plant Physiol 147: 1800-1804

Hammani K, Gobert A, Hleibieh K, Choulier L, Small I, Giege P (2011) An Arabidopsis dual-localized pentatricopeptide repeat protein interacts with nuclear proteins involved in gene expression regulation. Plant Cell 23: 730-740

Huang, C. Y., Ayliffe, M. A. & Timmis, J. N. 2003 Direct measurement of the transfer rate of chloroplast DNA into the nucleus. Nature 422, 72-76

Inze A, Vanderauwera S, Hoeberichts FA, Vandorpe M, Van Gaever T, Van Breusegem F (2012) A subcellular localization compendium of hydrogen peroxide-induced proteins. Plant Cell Environ 35: 308-320

Jacob Y, Feng S, LeBlanc CA, Bernatavichute YV, Stroud H, Cokus S, Johnson LM, Pellegrini M, Jacobsen SE, Michaels SD (2009) ATXR5 and ATXR6 are novel H3K27 monomethyltransferases required for chromatin structure and gene silencing. Nat Struct Mol Biol 16: 763-768

Jacob Y, Stroud H, LeBlanc C, Feng S, Zhuo L, Caro E, Hassel C, Gutierrez C, Michaels SD, Jacobsen SE (2010) Regulation of heterochromatic DNA replication by histone H3 lysine 27 methyltransferases. Nature 466: 987-991

Jacob Y, Bergamin E, Donoghue MTA, Mongeon V, LeBlanc C, Voigt P, Underwood CJ, Brunzelle JS, Michaels SD, Reinberg D, Couture JF, Martienssen RA (2014) Selective methylation of histone H3 variant H3.1 regulates heterochromatin replication. Science 343: 1249-1253

Jeong SY, Rose A, Meier I (2003) MFP1 is a thylakoidassociated, nucleoid-binding protein with a coiled-coil structure. Nucl Ac Res 31: 5175-5185

Jiang YH, Qian X, Shen JF, Wang YG, Li XJ, Liu R, Xia Y, Chen QM, Peng G, Lin SY, Lu ZM (2015) Local generation of fumarate promotes DNA repair through inhibition of histone H3 demethylation. Nature Cell Biol 17: 1158-+

König AC, Hartl M, Pham PA, Laxa M, Boersema PJ, Orwat A, Kalitventseva I, Plochinger M, Braun HP, Leister D, Mann M, Wachter A, Fernie AR, Finkemeier I (2014) The Arabidopsis class II sirtuin Is a lysine deacetylase and interacts with mitochondrial energy metabolism. Plant Physiol 164: 1401-1414

Kodama Y, Sano H (2006) Evolution of a basic helix-loop-helix protein from a transcriptional repressor to a plastid-resident regulatory factor: Involvement in hypersensitive cell death in tobacco plants. J Biol Chem 281: 35369–35380

Krause K, Kilbienski I, Mulisch M, Rödiger A, Schäfer A, Krupinska K (2005) DNA-binding proteins of the Whirly family in *Arabidopsis thaliana* are targeted to the organelles. FEBS Lett 579: 3707-3712

Krupinska K, Dähnhardt D, Fischer-Kilbienski I, Kucharewicz W, Scharrenberg C, Trösch M, Buck F (2014b) Identification of WHIRLY1 as a factor binding to the promoter of the stress- and senescence-associated gene H*vS4*0. J Plant Growth Reg 33: 91-105

Krupinska K, Oetke S, Desel C, Mulisch M, Schäfer A, Hollmann J, Kumlehn J, Hensel G (2014a) WHIRLY1 is a major organizer of chloroplast nucleoids. Fron Plant Sci 5: 432

Kupsch C, Ruwe H, Gusewski S,Tillich M, Small I, Schmitz-Linneweber C (2012) Arabidopsis chloroplast RNA binding proteins CP31A and CP29A associate with large transcript pools and confer cold stress tolerance by influencing multiple chloroplast RNA processing steps. Plant Cell 24: 4266-4280

Kwon C, Chung IK, (2004) Interaction of an Arabidopsis RNA-binding protein with plant single-stranded telomeric DNA modulates telomerase activity. J Biol Chem 279: 12812–12818

Lai ZB, Li Y, Wang F, Cheng Y, Fan BF, Yu JQ, Chen ZX (2011) Arabidopsis sigma factor binding proteins are activators of the WRKY33 transcription factor in plant defense. Plant Cell 23: 3824-3841

Lichocka M, Rymaszewski W, Morgiewicz K, Barymow-Filoniuk I, Chlebowski A, Sobczak M, Samuel MA, Schmelzer E, Krzymowska M, Hennig J (2018) Nucleus- and plastid-targeted annexin 5 promotes reproductive development in Arabidopsis and is essential for pollen and embryo formation. BMC Plant Cell Biol. 18: 183

Luo M, Orsi R, Patrucco E, Pancaldi S, Cella R. (1997) Multiple transcription start sites of the carrot dihydrofolate reductase-thymidylate synthase gene, and sub-cellular localization of the bifunctional protein. Plant Mol Biol 33: 709–722

Lv RQ, Li ZH, Li MP, Dogra V, Lv SS, Liu RY, Lee KP, Kim CH (2019) Uncoupled expression of nuclear and plastid photosynthesis-associated genes contributes to cell death in a lesion mimic mutant. Plant Cell 31: 210-230

Ma Z, Dooner HK (2004) A mutation in the nuclear-encoded plastid ribosomal protein S9 leads to early embryo lethality in maize. Plant J. 37: 92–103

Marti MC, Olmos E, Calvete JJ, Diaz I, Barranco-Medina S, Whelan J, Lazaro JJ, Sevilla F, Jimenez A (2009) Mitochondrial and nuclear localization of a novel pea thioredoxin: identification of its mitochondrial target proteins. Plant Physiol 150: 646-657

Meier I, Phelan T, Gruissem W, Spiker S, Schneider D (1996) MFP1, a novel plant filament-like protein with affinity for matrix attachment region DNA. Plant Cell 8: 2105–2115

Melonek J, Matros A, Trösch M, Mock HP, Krupinska K (2012) The core of chloroplast nucleoids contains architectural SWIB-domain proteins. Plant Cell 24: 3060-3073

Morikawa K, Shiina T, Murakami S, Toyoshima Y (2002) Novel nuclear-encoded proteins interacting with a plastid sigma factor, Sig1, in *Arabidopsis thaliana*. FEBS Letters 514: 300-304

Neuburger M, Rébeillé F, Jourdain A, Nakamura S, Douce R (1996) Mitochondria are a major site for folate and thymidylate synthesis in plants. J Biol Chem 271: 9466–9472

Ohta M, Sugita M, Sugiura M (1995) Three types of nuclear genes encoding chloroplast RNA-binding proteins (cp29, cp31 and cp33) are present in *Arabidopsis thaliana*: Presence of cp31 in chloroplasts and its homologue in nuclei/cytoplasms. Plant Mol Biol 27: 529–539

Pfalz J, Liere K, Kandlbinder A, Dietz KJ, Oelmüller R (2006) pTAC2, -6, and -12 are components of the transcriptionally active plastid chromosome that are required for plastid gene expression. Plant Cell 18: 176–197

Pfalz P, Holtzegel U, Barkan A, Weisheit W, Mittag M, Pfannschmidt T (2015) ZmpTAC12 binds single-stranded nucleic acids and is essential for accumulation of the plastid-encoded polymerase complex in maize. New Phytol 206: 1024-1037

Pfannschmidt T, Blanvillain R, Merendino L, Courtois F, Chevalier F, Liebers M, Grubler B, Hommel E, Lerbs-Mache S (2015) Plastid RNA polymerases: orchestration of enzymes with different evolutionary origins controls chloroplast biogenesis during the plant life cycle. J Exp Bot 66: 6957-6973

Powikrowska M, Khrouchtchova A, Martens HJ, Zygadlo-Nielsen A, Melonek J, Schulz A, Krupinska K, Rodermel S, Jensen PE (2014a) SVR4 (suppressor of variegation 4) and SVR4-like: two proteins with a role in proper organization of the chloroplast genetic machinery. Physiol Plant 150: 477-492

Qiao JW, Ma CL, Wimmelbacher M, Bornke F, Luo MZ (2011) Two novel proteins, MRL7 and its Paralog MRL7-L, have essential but functionally distinct roles in chloroplast development and are involved in plastid gene expression regulation in Arabidopsis. Plant Cell Physiol 52: 1017-1030

Qiu Y, Li M, Pasoreck EK, Long L, Shi Y, Galvão RM, Chou CL, Wang H, Sun AY, Zhang YC, Jiang A, Chen M (2015) HEMERA couples the proteolysis and transcriptional activity of PHYTOCHROME INTERACTING FACTORs in Arabidopsis photomorphogenesis. Plant Cell 27: 1409–1427

Raynaud C, Perennes C, Reuzeau C, Catrice O, Brown S, Bergounioux C (2005) Cell and plastid division are coordinated through the prereplication factor AtCDT1. Proc Natl Ac Sci USA 102: 8216-8221

Raynaud C, Sozzani R, Glab N, Domenichini S, Perennes C, Cella R, Kondorosi E, Bergounioux C (2006) Two cell-cycle regulated SET-domain proteins interact with proliferating cell nuclear antigen (PCNA) in Arabidopsis. Plant J 47: 395-407

Ruwe H, Kupsch C, Teubner M, Schmitz-Linneweber C (2011) The RNA-recognition motif in chloroplasts. J Plant Physiol 168: 1361–1371

Saelim L, Akiyoshi N, Tan TT, Ihara A, Yamaguchi M, Hirano K, Matsuoka M, Demura T, Ohtani M (2018) Arabidopsis Group IIId ERF proteins positively regulate primary cell wall-type CESA genes. J Plant Res 132: 117-129

Samaniego R, Jeong SY, Meier I, de la Espina SM (2006) Dual location of MAR-binding, filament-like protein 1 in Arabidopsis, tobacco, and tomato. Planta 223: 1201–1206

Sato N, Ohshima K, Watanabe A, Ohta N, Nishiyama Y, Joyard J, Douce R (1998) Molecular characterization of the PEND protein, a novel bZIP protein present in the envelope membrane that is the site of nucleoid replication in developing plastids. Plant Cell 10: 859–872

Schwacke R, Fischer K, Ketelsen B, Krupinska K, Krause, K (2007) Comparative survey of plastid and mitochondrial targeting properties of transcription factors in Arabidopsis and rice. Mol Genet Genom 277: 631–646

Seung D, Schreier TB, Bürgy L, Eicke S, Zeeman SC (2018) Two Plastidial Coiled-Coil Proteins Are Essential for Normal Starch Granule Initiation in Arabidopsis. Plant Cell 30: 1523-1542

Steiner S, Schröter Y, Pfalz J, Pfannschmidt T (2011) Identification of essential subunits in the plastid-encoded RNA polymerase complex revealsbuilding blocks for proper plastid development. Plant Physiol 157: 1043–1055

Sun TH, Zhou F, Liu CJ, Zhuang Z, Lu S (2016) The DnaJ-like zinc finger domain protein ORANGE localizes to the nucleus in etiolated cotyledons of *Arabidopsis thaliana*. Protoplasma 253: 1599-1604

Sun Q, Li YY, Wang Y, Zhao HH, Zhao TY, Zhang ZY, Li DW, Yu JL, Wang XB, Zhang YL, Han CG (2018) Brassica yellows virus P0 protein impairs the antiviral activity of NbRAF2 in Nicotiana benthamiana. J Exp Bot 69: 3127-3139

Sunderland, P., West, C., Waterworth, W. & Bray, C. 2006 An evolutionarily conserved translation initiation mechanism regulates nuclear or mitochondrial targeting of DNA ligase 1 in *Arabidopsis thaliana*. Plant J 47, 356-367.

Terasawa K, Sato N (2009) Plastid localization of the PEND protein is mediated by a noncanonical transit peptide. FEBS J 276: 1709–1719.

Waldmüller S, Müller U, Link G (1996) GSBF1, a seedling-specific bZIP DNA-binding protein with preference for a ‘split’ G-box-related element in Brassica napus RbcS promoters. Plant Mol Biol 32: 631–639.

Wang LS, Apel K (2019) Dose-dependent effects of O-1(2) in chloroplasts are determined by its timing and localization of production. Journal of Exp Bot 70: 29-40

Yang E, Yoo CY, Liu J, Wang H, Cao J, Li F-W, Pryer K, Sun T-P, D W, Zhou P, Chen M (2019) NCP activates chloroplast transcription by controlling phytochrome-dependent dual nuclear and plastidial switches. Nat Commun 10: 2630

Yoo HH, Kwon C, Lee MM, Chung IK (2007) Single-stranded DNA binding factor AtWHY1 modulates telomere length homeostasis in Arabidopsis. Plant J 49: 442-451

Yoo HH, Kwon C, Chung IK (2010) An Arabidopsis splicing RNP variant STEP1 regulates telomere length homeostasis by restricting access of nuclease and telomerase. Mol Cells 30: 279–283

Yoo CY, Pasoreck E, Wang H, Cao J, Blaha G, Weigel D, Chen M (2019) Phytochrome activates the plastid-encoded RNA polymerase for chloroplast biogenesis via nucleus-to-plastid signaling. Nature Comm 10: 2629

Zhou XJ, Welsch R, Yang Y, Alvarez D, Riediger M, Yuan H, Fish T, Liu JP, Thannhauser TW, Li L (2015) Arabidopsis OR proteins are the major posttranscriptional regulators of phytoene synthase in controlling carotenoid biosynthesis. Proc Nat Ac Sci USA 112: 3558-3563
